# “Eating and Nutrition Habits in Young Competitive Athletes: A Comparison Between Soccer Players and Cyclists”

**Published:** 2014-09-01

**Authors:** G. Galanti, L. Stefani, I. Scacciati, G. Mascherini, G. Buti, N. Maffulli

1. The address of Dr Stefani, corresponding author, is as follows:
  Laura Stefani
  Sport Medicine Center, University of Florence-Viale
  Morgagni 45, Florence-Italy
  Tel: 0039(055)7947066
  E-mail: laura.stefani@unifi.it
2. Tables cited in this paper are as follows:

**Table 1:** BIA estimated values in the normal range

**Body Water**	**Normal range**
TBW	>60%
ECW	>39%
BCM	>50%
FM	<18%

Legend: TBW: Total Body Water; ECW Extra Cellular Water; BCM Body Cellular Mass; FM Fat Mass (Bia Akern Srl Florence)

**Table 2.** Bioimpedance parameters of Soccer Players and Cyclists

	**Soccers Players** Mean±SD	**Cyclists** Mean±SD	**Normal range**
**FFM** (%)	85.4 ± 2.9	80.5 ± 3.5	
**FM (**%)	14.5 ± 2.9	19.4 ± 3.5	<18%
**PA (°)**	7.1 ± 0.5	7.3 ± 0.6	>6°; < 8°
**TBW (**%)	63.8± 1.9	59.8 ± 8.6	>60%
**ECW (**%)	43.8 ± 3.0	43.8 ± 2.1	>39%
**ICW (**%)	57.4 ± 1.8	56.1 ± 2.0	
**BCM (**%)	58.9 ± 2.2	56.5 ± 9.0	>50%
**BMR (**KCal)	1742.6 ± 156.2	1535.4 ± 193.1	

Legend: FFM Fat Free Mass; FM Fat Mass; PA Phase Angle; TBW Total Body Water; ICW IntraCellular Water; BCM Body Cellular Mass; BMR Basal Metabolic Rate; ST. DEV Standard Deviation

**Table 3.** Caloric distribution and quantities of protein, fat, carbohydrate, sugar, fiber, cholesterol and fatty acid distribution consumed on by the two groups of young athletes

	**Soccers** Mean±SD	**Cyclists** Mean±SD	LARN
Calories (Kcal)	2844.0 ± 51.4	2632.5 ± 923.8	
Alcol (Kcal)	9.9 ± 19.8	9.9 ± 39.8	
Protein (g)	115.8± 12.0 (15%)	97.7± 36.3 (16%)	15%
Fats (g)	118.7 ± 8.7 (34%)	84.4± 34.6 (29%)	20–35%
Carbohydrates (g)	429.1± 26.2 (51%)	391.4± 136.3 (55%)	55–65%
Oligosaccharides (g)	130.0± 13.0 (17%)	117.9± 37.9 (18%)	15/60%
Total fiber (g)	21.1 ± 2.2	23.0 ± 8.3	25–30
Cholesterol (mg)	252.1 ± 12.1	283.1 ± 136.6	300
Satured fatty acid (g)	34.25 ± 5.11	26.72 ± 11.5	7–10 %
Polyunnsatured fatty acid (g)	13.67 ± 0.29	12.85 ± 6.8	< 20%
Monunsatured fatty acid (g)	52.82 ± 4.41	40.22 ± 16.7	Up to 20%

Legend: LARN is an Italian acronyms corresponding to a recommended Reference Levels of Nutrients and energy for the Italian population (10)

**Table 4.** Comparison between minerals and vitamin intake and LARN recommendations (10). Quantities of minerals consumed and the corresponding percentage of the adequate intakes.

	**Soccer players** Mean±SD	**Cyclists** Mean±SD	LARN
**Calcium (Mg)**	1150.7 ± 128.9	718.1 ± 308.9	1300
**Sodium (Mg)**	2675.5 ± 399.9	2341.9 ± 1089.0	1500
**Potassium (Mg)**	2576.8 ± 52.4	3208.1 ± 1180.1	3900
**Phosphorus (Mg)**	1677.0 ± 98.5	1375.6 ± 478.9	1250
**Iron (Mg)**	11.1 ± 1.0	12.4 ± 4.5	10–13
**Zinc (Mg)**	10.0 ± 1.1	10.6 ± 4.3	9–11
**Folic Acid (Mcg)**	113.8 ± 0.2	352.8 ± 125.1	200
**Niacin (Mg)**	16.0 ± 1.3	22.3 ± 9.3	12–14
**Riboflavin (Mg)**	2.2 ± 0.1	2.7 ± 1.2	1.2–1.3
**Tiamin (Mg)**	1.30 ± 0.13	1.73 ± 0.64	1.2
**Vitamin ‘A’ (Mcg)**	792.0 ± 44.1	856.3 ± 822.3	300–450
**Vitamin ‘B6’ (Mg)**	0.8 ± 0.01	2.6 ± 1.0	1–1.1
**Vitamin ‘C’ (Mg)**	113.3 ± 17.7	141.7 ± 75.4	67–75
**Vitamin ‘D’ (Mg)**	1.5 ± 0.3	2.3 ± 1.7	0–1
**Vitamin ‘E’ (Mg)**	9.0 ± 0.2	13.2 ± 4.8	8

Legend: LARN is an Italian acronyms corresponding to a recommended Reference Levels of Nutrients and energy for the Italian population (10).

**Table 5:** caloric distribution of meals. Percentages of total energy intake at breakfast, morning snack, lunch, afternoon snack, dinner and evening snack.

	**Soccer** Mean±SD	**Cyclists** Mean±SD	Recommended Levels
**Breakfast**	10.4 ± 0.1	14.2 ± 6.8	20/25
**Snack**	6.7 ± 2.3	6.5 ± 6.1	10/15
**Lunch**	32.4 ± 1.0	29.3 ± 7.0	25/30
**Snack**	9.8 ± 2.4	7.7 ± 4.6	10/15
**Dinner**	34.9 ± 1.0	37.6 ± 8.9	30/35
**Snack**	5.4 ± 1.9	4.1 ± 2.5	5/10

